# Internalization of Titanium Dioxide Nanoparticles Is Cytotoxic for H9c2 Rat Cardiomyoblasts

**DOI:** 10.3390/molecules23081955

**Published:** 2018-08-06

**Authors:** Elizabeth Huerta-García, Iván Zepeda-Quiroz, Helen Sánchez-Barrera, Zaira Colín-Val, Ernesto Alfaro-Moreno, María del Pilar Ramos-Godinez, Rebeca López-Marure

**Affiliations:** 1 Departamento de Fisiología (Biología Celular), Instituto Nacional de Cardiología “Ignacio Chávez”, Juan Badiano No. 1, Colonia Sección XVI, Tlalpan, C.P. 14080, Ciudad de México, Mexico; marlon_32001@yahoo.com.mx (E.H.-G.); poke_621@hotmail.com (I.Z.-Q.); helenisimasab@gmail.com (H.S.-B.); zaira.cv.10@gmail.com (Z.C.-V.); 2 Swetox, Karolinska Institutet, Unit of Toxicology Sciences, Forskargatan 20, SE-151 36 Södertälje, Sweden; ernesto.alfaro-moreno@swetox.se; 3 Departamento de Microscopía Electrónica, Instituto Nacional de Cancerología, Av. San Fernando No. 22, Colonia Sección XVI, Tlalpan, C.P. 14080 Ciudad de México, Mexico; pilyrg@gmail.com

**Keywords:** titanium dioxide nanoparticles, cardiomyoblasts, internalization, oxidative stress, necrosis, autophagy

## Abstract

Titanium dioxide nanoparticles (TiO_2_ NPs) are widely used in industry and daily life. TiO_2_ NPs can penetrate into the body, translocate from the lungs into the circulation and come into contact with cardiac cells. In this work, we evaluated the toxicity of TiO_2_ NPs on H9c2 rat cardiomyoblasts. Internalization of TiO_2_ NPs and their effect on cell proliferation, viability, oxidative stress and cell death were assessed, as well as cell cycle alterations. Cellular uptake of TiO_2_ NPs reduced metabolic activity and cell proliferation and increased oxidative stress by 19-fold measured as H_2_DCFDA oxidation. TiO_2_ NPs disrupted the plasmatic membrane integrity and decreased the mitochondrial membrane potential. These cytotoxic effects were related with changes in the distribution of cell cycle phases resulting in necrotic death and autophagy. These findings suggest that TiO_2_ NPs exposure represents a potential health risk, particularly in the development of cardiovascular diseases via oxidative stress and cell death.

## 1. Introduction

Titanium dioxide nanoparticles (TiO_2_ NPs) are widely used in foods, medicines, and cosmetics [1]. Human exposure occurs either by oral, dermal or inhalation routes [2]. Although initially considered safe and inert, TiO_2_ NPs may actually be harmful for human health. For example, mice exposed to TiO_2_ NPs developed strong pulmonary inflammation, acute phase responses and cytokine release into circulation [3]. In mice instilled intratracheally with low (18 μg) and high (162 μg) TiO_2_ NPs doses, these nanoparticles were accumulated in heart and liver and translocated into circulation 24 h after exposure [4]. TiO_2_ NPs activated the complement cascade and inflammatory processes in the heart, and triggered early innate immune responses in blood mediated by the complement factor 3. In liver, TiO_2_ NPs altered gene expression related to acute phase response [4]. Oral and intravenous administrations of various TiO_2_ NPs resulted in accumulation in many organs including liver, lung and heart, regardless of particle size, crystalline form or hydrophobicity [5]. Furthermore, abdominal injection of TiO_2_ NPs in mice caused titanium accumulation in several organs, seriously damaging the liver, kidneys and heart and altering blood sugar and lipids [6]. Taken together, these results suggest that TiO_2_ NPs can accumulate in different organs producing tissue damage and inflammation.

Despite TiO_2_ NPs have been described as an inert material [7], several studies have shown the opposite. In the cardiovascular system, several experiments in vivo have shown myocardial damage, oxidative stress, inflammation and atherosclerosis in mice exposed to TiO_2_ NPs [6]. Daily gastrointestinal administration of TiO_2_ NPs at 0, 2, 10, 50 mg/kg in rats for up to three months resulted in cardiac dysfunction and inflammatory response [7]. Intragastric feeding of mice with TiO_2_ NPs for nine consecutive months resulted in their accumulation in the heart causing inflammation, apoptosis and cardiac dysfunction [8].

Other studies have shown a strong increase of reactive oxygen species (ROS). Sheng and collaborators [9] administered different doses (2.5, 5, 10 mg/kg body weight) of TiO_2_ NPs for a long-term exposure (90 days) inducing oxidative stress and antioxidant system attenuation in mice heart. TiO_2_ NPs accumulated in the heart causing sparse cardiac muscle fibers, inflammatory response, cell necrosis, and cardiac biochemical dysfunction. There was an increase of superoxide radicals, hydrogen peroxide, malondialdehyde, carbonyl and 8-OHdG, as well as degradation products of proteins, lipids and DNA oxidation [9]. Overall, cell toxicity induced by nanoparticles has been associated to induction of oxidative stress. One study showed that macrophage-like THP-1 and HPMEC-ST1.6R microvascular cells exposed to TiO_2_ NPs were sensitive to endogenous redox changes and apoptosis [10,11]. A549 cells incubated with TiO_2_ NPs (Anatase 22.1 nm) for 24 h showed reduced cell viability and increased lactate dehydrogenase activity in a concentration-dependent manner, indicating cell membrane damage [12]. Also murine microglial cells (BV-2) treated at different concentrations of TiO_2_ NPs (0.1 to 200 μg/mL) showed a slight inhibition of cell growth. High TiO_2_ NPs concentrations enhanced permeability of cytoplasmic membrane to propidium iodide (PI), associated with loss of mitochondrial membrane potential (ΔΨm) and overproduction of superoxide anions [13]. In primary rat cortical astrocytes and human lung fibroblast cells (WI-38), TiO_2_ NPs induced ROS generation and reduced ΔΨm [14]. Moreover, food grade TiO_2_ NPs promoted intracellular oxidative stress in WI-38 cells, altering cell cycle progression (G2/M > S > G0/G1) [15]. 

Many studies have described negative effects of TiO_2_ NPs in various systems and cell types [2,11]. In the cardiovascular system, these nanoparticles induce tissue damage and inflammatory responses; however, the underlying mechanisms are not well understood. Therefore, in order to assess the impact of TiO_2_ NPs on cardiac cells we evaluated their cellular uptake in H9c2 rat cardiomyoblasts and the mechanisms associated with their nanotoxicity.

Since TiO_2_ NPs can translocate into the systemic circulation and the heart [4], we hypothesized that these nanoparticles could induce damage to cardiac cells. To test this hypothesis, we exposed H9c2 cells to TiO_2_ NPs and examined their effects on cell cycle phases, mitochondrial function, oxidative stress, cell death and autophagy.

## 2. Results

### 2.1. Internalization of TiO_2_ NPs

**Figure 1:**
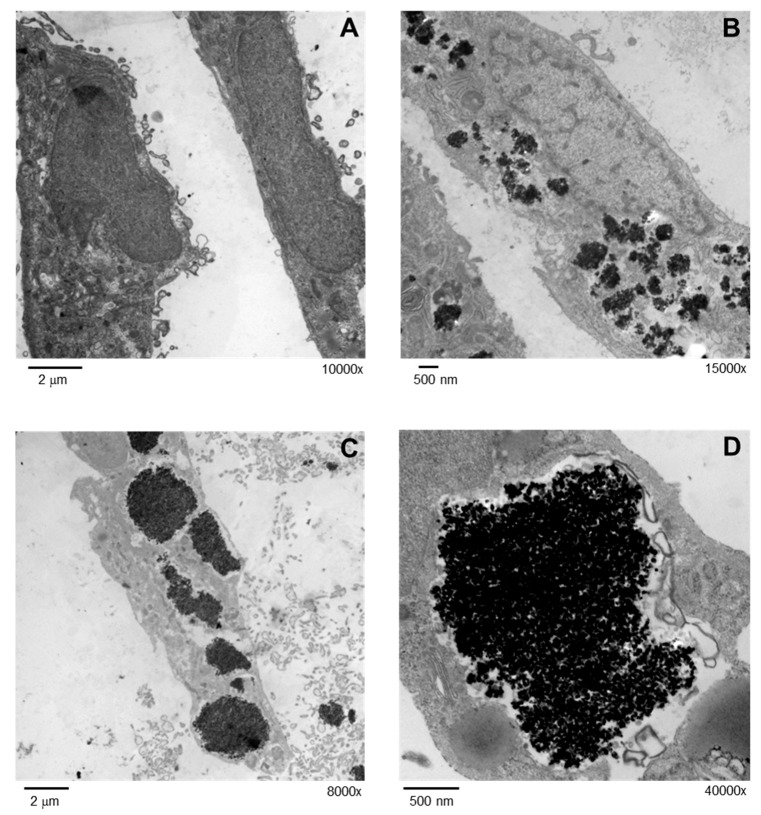
Internalization of TiO_2_ NPs was evaluated by TEM. Cells were treated with 5 μg/cm^2^ TiO_2_ NPs for 24 h and analyzed in a JEOL 10-10 microscope and an AMT Camera System. TEM micrographs of non-exposed cells at a direct magnification of 10,000× (**A**) and treated cells a magnification of 15,000× (**B**), 8000× (**C**) and 40,000× (**D**) are shown.

Due to their small size, nanoparticles uptake can occur in cardiac cells. In order to corroborate this, H9c2 cells were exposed to 5 μg/cm^2^ TiO_2_ NPs for 24 h and were then analyzed by transmission electron microscopy (TEM). Numerous nanoparticle aggregates with size < 500 nm were observed inside cells (Figure 1B); however, large aggregates > 2 μM were also present (Figure 1C,D). Internalized TiO_2_ NPs were localized in the cytoplasm but solid core NPs were not observed inside cell organelles.

### 2.2. TiO_2_ NPs Inhibited Proliferation and Decreased Metabolic Activity

**Figure 2:**
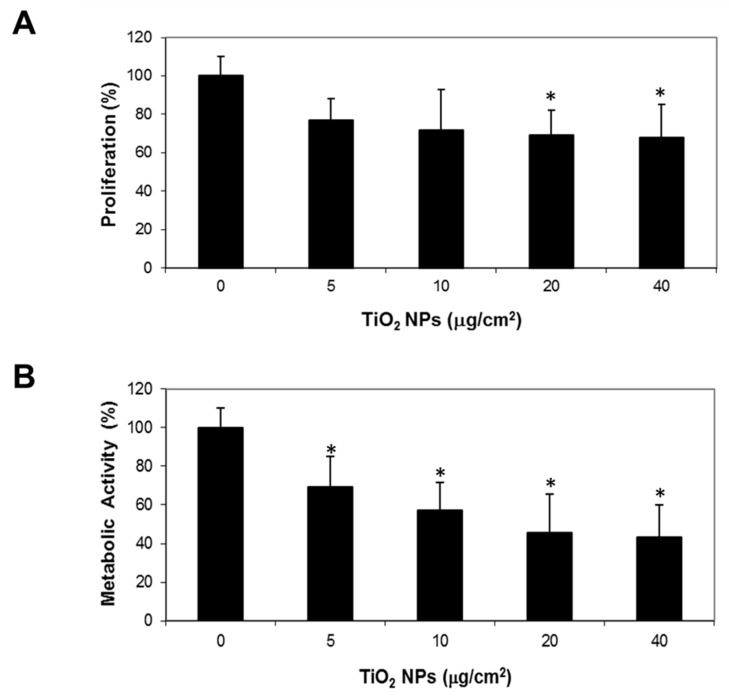
TiO_2_ NPs treatment inhibited cell proliferation and decreased metabolic activity. H9c2 cells were treated with different TiO_2_ NPs concentrations (5, 10, 20, 40 μg/cm^2^) for 48 h. Cell proliferation was evaluated by crystal violet staining and viability by MTT reduction. Results were expressed as mean ± standard deviation (SD) of three independent experiments (*n* = 15). * Significant difference between control (untreated) and treated cells (*p* < 0.05).

Large TiO_2_ NPs aggregates observed inside cells could induce cytostatic/cytotoxic effects, therefore we evaluated their impact on cell proliferation and viability. To measure proliferation, H9c2 cells were exposed to different TiO_2_ NPs concentrations for 72 h and were stained with crystal violet. Results showed that high NPs concentrations (20 and 40 μg/cm^2^) decreased cell proliferation in about 30% (*p* < 0.05 versus control cells) (Figure 2A). To evaluate viability, a MTT assay was performed. The metabolic activity was measured by MTT reduction to purple formazan by mitochondrial dehydrogenases in living cells. TiO_2_ NPs from 5 μg/cm^2^ decreased cell metabolic activity by 30%, and the maximum effect was achieved at 40 μg/cm^2^ with 60% inhibition, compared to control cells (Figure 2B). The half maximal inhibitory concentration (IC_50_) was 20 μg/cm^2^ (100 μg/mL); therefore, further experiments in H9c2 cells were performed at this concentration.

### 2.3. TiO_2_ NPs Changed Cellular Redox State

**Figure 3:**
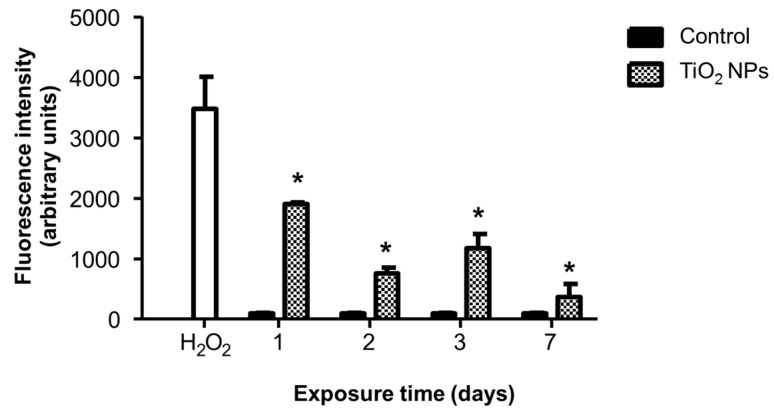
TiO_2_ NPs treatment changed cellular redox state. H9c2 cells were treated with TiO_2_ NPs (20 μg/cm^2^) alone for 1, 2, 3, and 7 days and cellular redox state was evaluated by H_2_DCFDA oxidation. Cells treated with H_2_O_2_ (500 μM) for 1 day were used as positive controls. Results were expressed as fluorescence intensity in arbitrary units and as mean ± standard deviation (SD) of three independent experiments (*n* = 15). * Significant difference between control (untreated) and treated cells (*p* < 0.05).

TiO_2_ NPs diminished cell viability and this cytotoxic effect is generally associated with oxidative stress. Therefore, we measured cellular redox state and ROS production by 2′,7′-dichlorodihydrofluorescein diacetate (H_2_DCFDA) oxidation. Results showed that TiO_2_ NPs strongly increased the fluorescence intensity in direct proportion to H_2_DCFDA oxidation. This increment was observed at all evaluated times; however, the highest effect was obtained at day one of treatment with a 17-fold increase (*p* > 0.05) vs. control cells (Figure 3).

### 2.4. TiO_2_ NPs Decreased the Mitochondrial Membrane Potential

**Figure 4:**
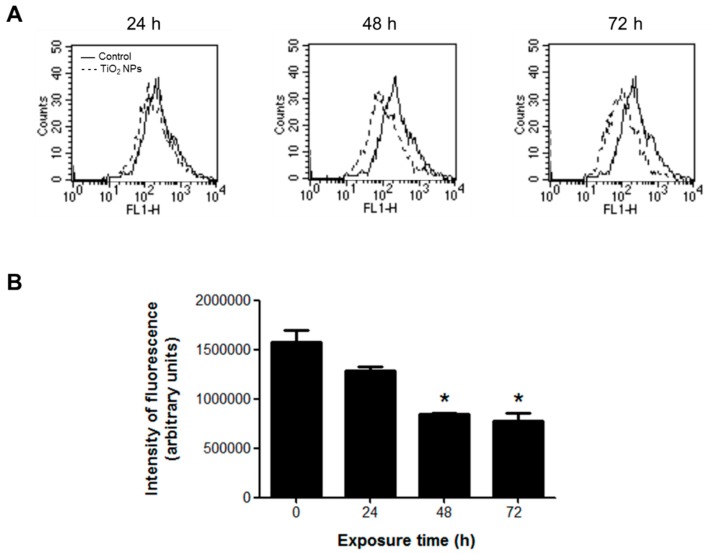
TiO_2_ NPs decreased ΔΨm in H9c2 cells treated with 20 μg/cm^2^ TiO_2_ NPs for 24, 48, and 72 h. ΔΨm changes were measured by the fluorescent dye Rh123 in a flow cytometer. (**A**) Histograms of a representative experiment performed independently. (**B**) Densitometric analysis expressed as fluorescence intensity (arbitrary units). Data are presented as mean ± standard deviation (SD) of three independent experiments (*n* = 3). * Significant difference between control (untreated) and treated cells (*p* < 0.05).

Oxidative stress was measured by changes in the ΔΨm with rhodamine 123 (Rh123). This molecule is cell membrane permeable and localizes in the mitochondria of viable cells, but when the ΔΨm is altered, Rh123 is released and the fluorescence intensity decreases. TiO_2_ NPs decreased the fluorescence by 50% with a significant statistical difference from 48 h of treatment, indicating alterations in the ΔΨm (Figure 4).

### 2.5. TiO_2_ NPs Altered Cell Cycle Phases

**Figure 5:**
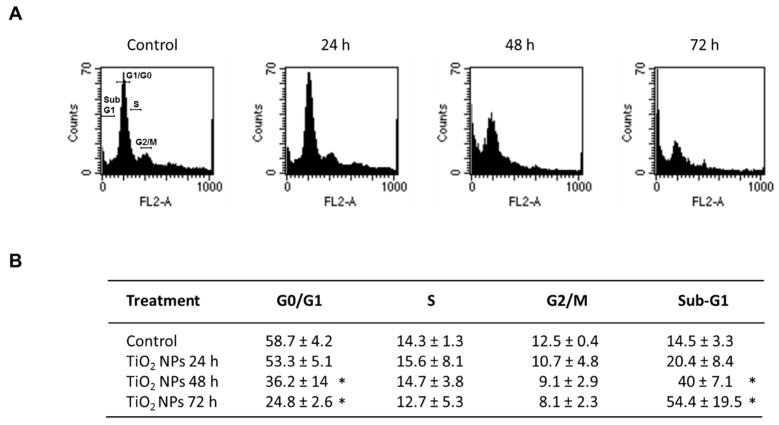
Effect of TiO_2_ NPs on cell cycle. H9c2 cells were treated with 20 μg/cm^2^ TiO_2_ NPs for 24, 48, and 72 h and cell cycle was analyzed by quantitation of DNA content through flow cytometry. Histograms (**A**) and table (**B**) show the percentage of cell populations in each phase. In (**B**), data were analyzed by the CellQuest Pro software (Becton Dickinson) and expressed as mean ± standard deviation (SD) of three independent experiments (*n* = 3). * Significant difference between control (untreated) and treated cells (*p* < 0.05).

To determine whether the effect of TiO_2_ NPs on cell proliferation and viability was associated with cell cycle alterations, H9c2 cells were exposed to 20 μg/cm^2^ TiO_2_ NPs for 24, 48 and 72 h and the cell cycle phases were evaluated. The number of cells in the G1 phase decreased by 22% after 48 h of treatment and reached 34% at 72 h compared with control cells. No significant changes were observed in the S and G2/M phases in the same periods. The percentages of sub G1 cells significantly increased in a time-dependent manner, and peaked at 72 h with 39.9%. These results indicate that NPs caused important changes in the distribution of cell cycle phases after 48 h of exposure (Figure 5).

### 2.6. TiO_2_ NPs Induced Necrotic Death and Autophagy

**Figure 6:**
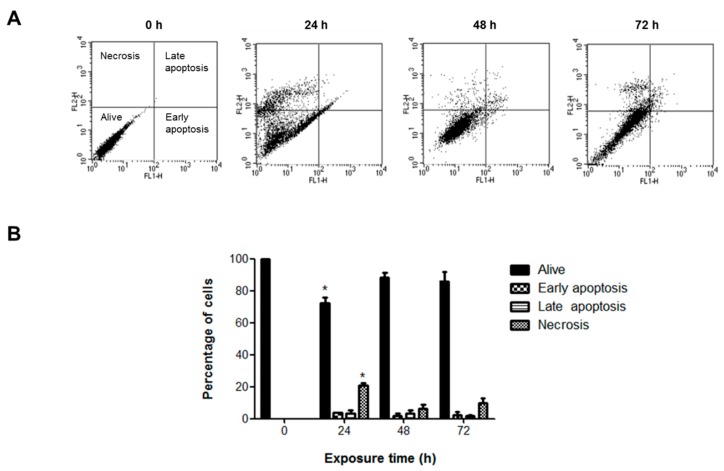
TiO_2_ NPs induced necrotic death. H9c2 cells were treated with 20 μg/cm^2^ TiO_2_ NPs for 24, 48, and 72 h, then apoptotic and necrotic death was measured by annexin-V and propidium iodide staining. Dot blots show resolution of live, apoptotic and necrotic populations (**A**) and the bar chart show the percentages (**B**) as mean ± standard deviation (SD) of three independent experiments (*n* = 3). * Significant difference between control (untreated) and treated cells (*p* < 0.05).

**Figure 7:**
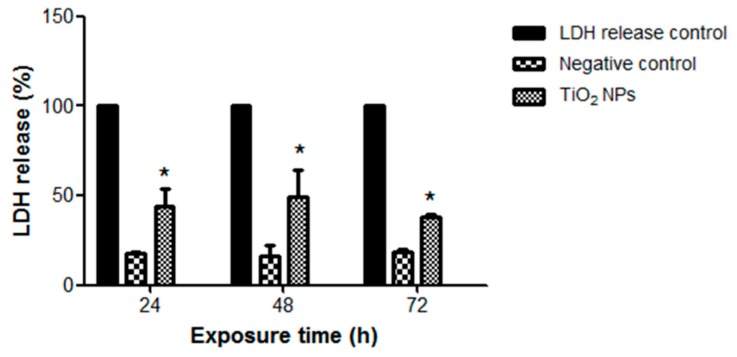
TiO_2_ NPs induced LDH release. H9c2 cells were treated with 20 μg/cm^2^ TiO_2_ NPs for 24, 48, and 72 h and then an LDH-based cytotoxicity assay was performed. Results are presented as mean ± standard deviation (SD) of three independent experiments (*n* = 3). * Significant difference between negative control (untreated) and treated cells (*p* < 0.05).

Since TiO_2_ NPs induced a significant increase in sub-G1 peak, we characterized the type of cell death. No significant change in apoptosis was observed with TiO_2_ NPs at any time; however, TiO_2_ NPs produced a slight but significant 20% increase in necrotic cell death after 24 h of treatment (Figure 6). This was consistent with higher LDH release (30%) (*p* < 0.05) at 24 and 48 h (Figure 7). 

**Figure 8:**
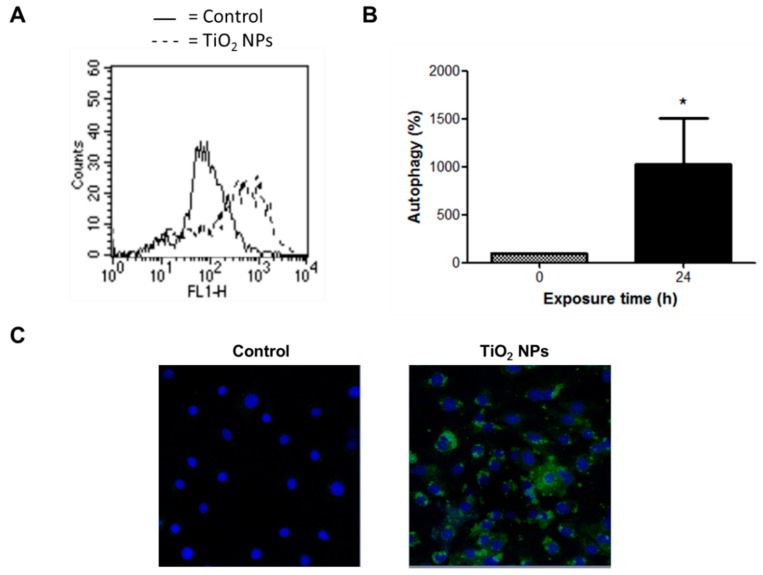
TiO_2_ NPs induced autophagy. H9c2 cells were treated with 20 μg/cm^2^ TiO_2_ NPs for 24 h and autophagy was evaluated through a detection kit by flow cytometry (**A**,**B**) and confocal microscopy (**C**). In (**B**), results are presented as mean ± standard deviation (SD) of three independent experiments (*n* = 3). * Significant difference between untreated cells (0) and TiO_2_ NPs-treated cells (*p* < 0.05). In (**C**), nuclear stain with DAPI and green detection reagent (autophagy) are showed.

TiO_2_ NPs also induced strong morphological changes related to increased numbers of cell vacuoles (data not shown). Since autophagy is a self-degradative process and a survival mechanism involving generation of vacuoles [16], autophagic vesicles were detected using a novel and selective green fluorescent dye. TiO_2_ NPs induced a nine-fold increase of fluorescence after 24 h of treatment, indicating the formation of autophagic vacuoles (Figure 8).

## 3. Discussion

There is evidence that TiO_2_ NPs can translocate to the heart via systemic circulation [4]. In this work we evaluated whether TiO_2_ NPs could have adverse effects on cardiomyocytes, measured through cell viability, oxidative stress, ΔΨm, cell cycle and cell death.

TiO_2_ NPs decreased cell proliferation and induced a strong cytotoxic effect on H9c2 cells, associated with increased oxidative stress and alterations of ΔΨm. Important changes in cell cycle phases were observed in association with necrotic death and autophagy. TiO_2_ NPs also disrupted the integrity of cell membrane leading to increased permeability and LDH release (Figure 6).

Despite the fact TiO_2_ NPs have been considered as inert and nontoxic, a growing body of evidence suggests quite the opposite. Cytotoxic effects of TiO_2_ NPs are generally associated with cell growth inhibition in different cells types [13,17,18,19,20,21,22]; however, our results are the first evidence of their toxicity in cardiac H9c2 cells. The inhibitory concentration IC_50_ was 20 μg/cm^2^ (75 μg/mL), consistent with other in vitro studies [23]. Although this concentration of TiO_2_ NPs is higher than those of occupational exposure or commercial products, TiO_2_ NPs may accumulate by long-term exposure and become toxic. Particles of few nanometers in size can translocate through the air-blood-barrier in approximately 10% [24]. Considering that 40 μg/cm^2^ of TiO_2_ NPs could be present in hot-spots of airways and lungs of exposed humans [25], then approximately 4 µg/cm^2^ could enter into systemic circulation. Once there, particles become highly diluted, but their bioaccumulation in different tissues is not well documented, therefore we hypothesized that these concentrations could be reached over long-term exposures. In a recent study, the biokinetics of 48 V-radiolabeled TiO_2_ NPs was investigated in rats at retention time points 1, 4, 24 h and seven days after oral application of a single dose by intra-esophageal instillation. Their results showed that 0.6% of the administered dose passed the gastro-intestinal-barrier after one hour and about 0.05% was still distributed in the body after seven days, indicating the possibility of chronic accumulation of nanoparticles in secondary organs and the skeleton [26].

We observed internalization of TiO_2_ NPs by H9c2 cells and these nanoparticles remained within cells even after cell division. Nanoparticles were accumulated in the cytoplasm but no interactions with organelles were observed (Figure 1). Chronic exposures at low concentrations of TiO_2_ NPs in human bronchial epithelium cells (BEAS-2B) showed cellular uptake and cell transformation [27], supporting our observations. Few studies have analyzed the exocytosis of nanoparticles in mammalian cells. Wang et al. [28] showed that TiO_2_ NPs were internalized by the neural stem cells after 48 h incubation, and only 35% was exocytosed after 24 h.

Nanoparticles interact directly with cells as complexes or aggregates [29]. The real identity and toxicity of TiO_2_ NPs in biological systems is a function of surface charge, size, solubility, shape, hydrophobicity, dose and crystalline structures [30]. Nanoparticle surface becomes saturated by phospholipids, proteins, DNA, small molecules and inorganic ions. The nanoparticle surface ligand induces protein corona misfolding and therefore indirectly enhances cellular uptake [31].

TiO_2_ reduction to nanosize increases surface area changing their electronic configuration and reactivity. These modifications also affect cell binding and internalization. The size of TiO_2_ NPs aggregates in culture medium containing FBS is reduced, enhancing dispersion [32], facilitating contact with cells and toxicity.

Our results showed that TiO_2_ NPs induced oxidative stress in H9c2 cells evidenced by changes in redox state (increased ROS production). After one day of exposure, TiO_2_ NPs induced ROS generation but this declined after two days. A subsequent increase and decrease occurred at three and seven days, respectively. We hypothesize that after two days of treatment, the antioxidant defense system counteracts cell and mitochondrial damage, but after three days, cells lose this capacity. After seven days of exposure, the extent of cell damage is greater, making difficult to evaluate ROS production. TiO_2_ NPs can produce ROS such as hydroxyl radicals and superoxides in the dark. These oxidize serum proteins to form a protein corona on the nanoparticles surface. This oxidized protein could be responsible for the oxidative stress induced by TiO_2_ NPs in H9c2 cells [33]. In a previous experiment performed in acellular conditions using a dithiothreitol (DTT) assay [34], we found the oxidant potential of TiO_2_ NPs.

Oxidative stress appears to be the underlying mechanism of in vivo genotoxicity of titanium [35]. We previously showed that oxidative stress induced by TiO_2_ NPs can upregulate early and late receptors for adhesion molecules on monocytes [36]. Taken together, these data indicate that oxidative stress plays an important role and could be the primary mechanism for TiO_2_ NPs toxicity in cardiomyoblasts.

Oxidative stress was related with dissipation of ΔΨm in H9c2 cells, indicating mitochondrial dysfunction. Similar results were observed in H9c2 cells exposed to platinum-coated TiO_2_ NPs (Pt-TiO_2_ NPs) [37]; and in primary astrocytes exposed to different types of TiO_2_ NPs, altering mitochondrial morphology, ROS generation, and ΔΨm, suggesting mitochondrial damage [14]. Mitochondrial dysfunction leads to ROS overproduction, damage to cellular components and cell death, forming a vicious cycle [38].

Studies in yeast and complex eukaryotes show that fluctuations in oxygen consumption, energy metabolism, and cell redox state are intimately integrated with cell cycle progression [39]. Therefore, we evaluated cell cycle phases in H9c2 cells exposed to TiO_2_ NPs. Our results showed that TiO_2_ NPs induced changes in the cell cycle. The proportion of G0/G1 phase cells decreased and the percentage of sub-G1 region events increased after 48 and 72 h of exposure, associated with necrosis and autophagy. Different forms of TiO_2_ NPs induced cell cycle arrest in various cells types [15,40,41], in connection with elevated ROS levels [40,42,43], indicating that cytotoxic effects of TiO_2_ NPs are related to oxidative stress, cell cycle alterations and cell death. 

H9c2 cells exposed to TiO_2_ NPs had severe damage resulting in strong autophagy (Figure 8). Autophagy involves lysosomal degradation of cytoplasmic components such as mitochondria and other intra-cellular structures [44,45]. Autophagy increases following mitochondrial dysfunction such as generation of low ATP levels; therefore, mitochondria have a key role in autophagy [46]. Autophagy is implicated in tumor suppression through cell cycle arrest, promoting genome and organelle integrity, or through inhibition of necrosis-mediated inflammation [16]. Autophagy has also been linked to pathologic conditions of cardiac remodeling that involve an increase of cardiomyocyte death [47,48,49,50]. Autophagy observed in H9c2 cells may be a consequence of necrosis and inflammation induced by TiO_2_ NPs in order to counteract the damage. In the heart autophagy can be either beneficial or harmful, but enhanced autophagy can induce cell death [51].

Some degree of necrosis but not apoptosis was observed in H9c2 cells exposed to TiO_2_ NPs. Cardiomyocytes may undergo apoptosis, necrosis and autophagic death [52]. Necrosis and apoptotic cell death depend in part, on ATP levels. In situations where ATP depletion is extreme, apoptosis is inhibited and then necrosis might occur [52]. Necrosis may also result from acidosis and higher calcium concentrations. Nanoparticles possibly induced changes in ATP levels, acidosis or increased intracellular calcium levels resulting in necrotic death of H9c2 cells.

The mitochondrial intermembrane protein and activator of caspases is released from the intermembrane space following outer membrane rupture. ROS production and mitochondrial alterations induced by TiO_2_ NPs in H9c2 cells may promote mitochondrial permeability transition and subsequent cell death. Cellular features of necrosis, apoptosis, and autophagy frequently co-occur after death signals and toxic stress [50]. Further studies are needed to evaluate whether long term exposure of cells to TiO_2_ NPs produces autophagic death.

**Figure 9:**
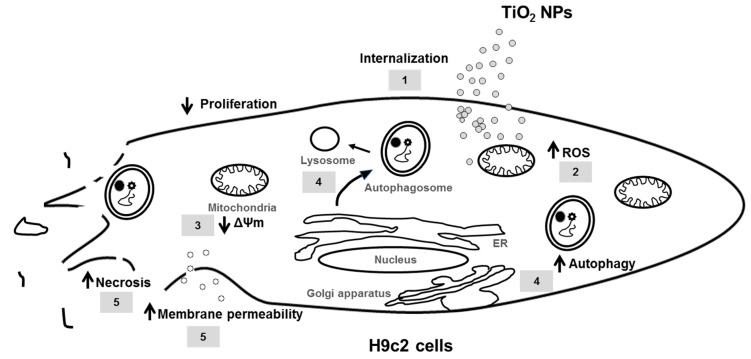
Toxic effects induced by TiO_2_ NPs in H9c2 cells. TiO_2_ NPs are internalized into the cytoplasm (1), producing strong oxidative stress and changes in the cellular redox state, increasing ROS production and mitochondrial damage (2), decreasing mitochondrial membrane potential (ΔΨm) (3). High oxidative stress causes strong cell damage, associated with autophagy (4), involving the formation of autophagosomes in the endoplasmic reticulum (ER), these in turn fuse with lysosomes to produce degradation of cytoplasmic components formed during cell damage. Finally, TiO_2_ NPs increase membrane permeability and induce necrosis (5).

In summary, TiO_2_ NPs cause severe damage to cardiomyoblasts cells *in vitro* through inhibition of proliferation (1), induction of oxidative stress and mitochondrial dysfunction (2, 3), autophagy (4), membrane permeability and necrotic death (5) (Figure 9), indicating that occupational and environmental exposures to these NPs, could eventually lead to heart damage and the development of cardiovascular diseases. Taken together, these results suggest that nanoparticles accumulation in cardiomyoblasts, could eventually drive cardiac damage and adverse health effects in the exposed population.

## 4. Methods

### 4.1. Materials

Dulbecco’s modified Eagle’s medium (DMEM) high glucose, 0.25% trypsin-EDTA solution, Antibiotic Antimycotic Solution (100×), and fetal bovine serum (FBS) were acquired from Gibco BRL (Grand Island, NY, USA). Cell culture consumables were purchased from Corning (Corning, NY, USA). Flow cytometry reagents were provided by Becton-Dickinson Immunocytometry Systems (San Jose, CA, USA). H_2_DCFDA was purchased from Molecular Probes, Invitrogen (Carlsbad, CA, USA). CytoTox 96 Non-radioactive cytotoxicity assay was from Promega (Madison, WI, USA). Western blot reagents were from Bio-Rad (Hercules, CA, USA). Autophagy detection kit was purchased from abcam (Cambridge, MA, USA). Anatase TiO_2_ NPs 25 nm and other chemicals were obtained from Sigma-Aldrich (St. Louis, MO, USA).

### 4.2. Culture of Embryonic Rat H9c2 Cardiomyoblast Cells

H9c2 rat cardiomyoblasts were used as a model since they mimic the hypertrophic responses of primary rat neonatal cardiomyocytes in vitro [53]. H9c2 cells were purchased from the American Type Culture Collection (CRL-1446, ATCC, Manassas, VA, USA) and cultured with DMEM high glucose added with 10% fetal bovine serum (FBS) plus an antibiotic-antimycotic solution. Cells were cultured at 37 °C in a humidified atmosphere of 5% CO_2_.

### 4.3. Titanium Dioxide Nanoparticles

TiO_2_ NPs were previously characterized by our group [54]. TiO_2_ NPs have a surface area of 45–50 m^2^/g with average particle size of 19 nm and ζ-potential of −12 mV. TiO_2_ NPs were endotoxin-free and pure, containing only oxygen and titanium [55]. Before use, TiO_2_ NPs were suspended at 1 mg/mL, in a HEPES phosphate buffer solution (HPBS: 4.4 mM KCl, 150 mM NaCl, 12.2 mM glucose, 10.9 mM HEPES, pH 7.4) and were vortexed a high speed for 2 min [54]. In previous studies, TiO_2_ NPs induced different toxic effects in a range from 1 to 100 μg/cm^2^. We also found that 40 μg/cm^2^ TiO_2_ NPs induced a strong cytotoxicity in other cells; therefore, in this work we tested concentrations equal or below this value (1, 5, 10, 20, 40 μg/cm^2^, equivalent to 5, 25, 50, 100 and 200 μg/mL). Concentrations are presented as μg/cm^2^ since TiO_2_ NPs suspensions are unstable and precipitate.

### 4.4. Internalization of TiO_2_ NPs

Cellular uptake of nanoparticles was evaluated by TEM as previously described by Huerta-García and collaborators [55]. Cells (200 × 10^3^/well) were treated with 5 μg/cm^2^ TiO_2_ NPs for 24 h. Then cells were fixed with 2.5% glutaraldehyde-formaldehyde in HPBS for 1 h. A second fixation was performed in 2% OsO_4_ (1:1 in HPBS) for 1 h. Cells were gradually dehydrated with increasing ethanol concentrations and embedded in epoxy resin (Epon 812, Sigma-Aldrich, St. Louis, MO, USA). Ultrathin sections were stained with lead citrate and alcoholic uranyl acetate. Finally, cells were examined with a transmission electron microscope (JEOL 10/10, MA, USA).

### 4.5. Proliferation Assay

H9c2 cells (8 × 10^3^ cells/well) were exposed to different concentrations of TiO_2_ NPs (5, 10, 20, 40 μg/cm^2^) and cell proliferation was evaluated by crystal violet staining after 72 h of treatment according to Márquez-Ramírez and collaborators [56].

### 4.6. Cell Viability

The reduction of 3-(4,5-dimethylthiazol-2-yl)-2,5-diphenyltetrazolium bromide (MTT) to water-insoluble formazan was used to evaluate cell viability. H9c2 cells (8 × 10^3^ cells/well) were exposed to different concentrations of TiO_2_ NPs (5, 10, 20, 40 μg/cm^2^) for 72 h. After treatment, cells were incubated with 5 mg/mL MTT for 4 h and optical density at 570 nm was measured in a microplate spectrophotometer.

### 4.7. Oxidative Stress

The cellular redox state and oxidative stress were measured by oxidation of the H_2_DCFDA (non-fluorescent) to 2′,7′-dichlorofluorescein (DCF) (highly fluorescent). Changes in the ΔΨm were assessed as described by Huerta-García and collaborators [57] using rhodamine 123 (Rh123), a cell-permeant cationic compound captured by the active mitochondria. H9c2 cells (1 × 10^6^ cells/treatment) were exposed to 20 μg/cm^2^ TiO_2_ NPs for 24, 48 and 72 h. After treatment, cell suspensions were incubated with 10 μM H_2_DCFDA or 0.2 μg/mL Rh123 for 30 min in the dark. Finally, cells were analyzed in a flow cytometer (Fascalibur, Becton Dickinson, Franklin Lakes, NJ, USA).

### 4.8. Cell Cycle Phases

Flow cytometry and staining with PI was performed to study cell cycle changes induced by TiO_2_ NPs. We selected the optimal concentration of TiO_2_ NPs for a significant reduction in cell proliferation. Therefore, H9c2 cells were exposed to 20 μg/cm^2^ TiO_2_ NPs for 24, 48 and 72 h. Then cells were fixed with 70% ethanol, washed with HPBS and incubated with RNAse (50 U/mL) for 1 h at 37 °C. Finally, cells were stained with PI (200 μg/mL) and analyzed by flow cytometry.

### 4.9. Cell Death

Apoptotic and necrotic death were measured by Annexin-V/PI staining and analyzed by flow cytometry. Necrosis was also assessed by lactate dehydrogenase (LDH) release. H9c2 cells were cultured with 20 μg/cm^2^ TiO_2_ NPs for 24, 48 and 72 h, incubated with 100 μL of Annexin-V plus PI in the dark at 37 °C for 30 min and then examined in a flow cytometer. To evaluate LDH release, cells were cultured in phenol red-free DMEM medium and exposed to nanoparticles. After exposure, 50 μL of the supernatant were mixed with 50 μL of the substrate mix and incubated in the dark at room temperature for 30 min. After incubation, 50 μL of stop solution were added and optical density was measured at 490 nm (OD_490_). Data were normalized by subtracting average background of culture medium from experimental values. Percentage of cytotoxicity was calculated by the formula:

Cytotoxicity (%) = (Experimental LDH release (OD_490_))/(Maximum LDH release control (OD_490_)) × 100

### 4.10. Autophagy

Autophagy was evaluated with a detection kit according to the manufacturer’s instructions. Autophagic vesicles are detected with a 488 nm-excitable green fluorescent dye and co-localization with LC3, a specific autophagosome marker. Cells were exposed to 20 μg/cm^2^ TiO_2_ NPs for 24 h. After exposure, cells were trypsinized, centrifuged at 1000 rpm for 5 min, and washed with 1× assay buffer. Then cells were centrifuged and re-suspended in 250 μL of phenol red-free cell culture medium containing 5% FBS and incubated with 250 μL of diluted green stain solution for 30 min at room temperature in the dark. After incubation, cells were collected by centrifugation, washed with 1× assay buffer and analyzed in a flow cytometer. To analyze autophagy by confocal microscopy, cells were grown on coverslips. After treatment with TiO_2_ NPs, medium was removed and cells were washed twice with 1× assay buffer. Then, cells were covered with 100 μL of microscopy dual detection reagent and incubated at 37 °C for 30 min in the dark. Then, cells were washed with 100 μL of 1× assay buffer, fixed with 4% formaldehyde and washed again three times. Finally, stained cells were analyzed with a model LSM 700 confocal microscope (Zeiss, Thornwood, NY, USA).

### 4.11. Statistical Analysis

Data are presented as mean ± standard deviation (SD) of at least three independent experiments. Data were analyzed by one-way analysis of variance (ANOVA) followed by post-hoc Tukey’s multiple comparison test, using GraphPad prism software version 5.01 (GraphPad Software La Jolla, CA, USA). Differences among groups were considered statistically significant at *p* < 0.05.
